# Outcome of People with Parkinson’s Disease Treated with Levodopa-Entacapone-Carbidopa Intestinal Gel Who Failed Previous Subcutaneous Foslevodopa/Foscarbidopa

**DOI:** 10.3390/brainsci16030343

**Published:** 2026-03-22

**Authors:** Diego Santos García, Inés Legarda, Tamara M. González Fernández, Ana Rodríguez Sanz, Maria Isabel Morales-Casado, Alejandro Peral, Nuria Caballol, María Álvarez Sauco, Iria Campos Rodríguez, Déborah Alonso Modino, Lydia López Manzanares, Jesús Olivares Romero, Alberto Blanco Ollero

**Affiliations:** 1 Department of Neurology, Hospital Universitario de A Coruña (HUAC), Complejo Hospitalario Universitario de A Coruña (CHUAC), C/As Xubias 84, 15006 A Coruña, Spain; 2 Grupo de Investigación en Enfermedad de Parkinson y otros Trastornos del Movimiento, INIBIC (Instituto de Investigación Biomédica de A Coruña), 15006 A Coruña, Spain; 3 Hospital San Rafael, 15006 A Coruña, Spain; 4 Fundación Degen, 15004 A Coruña, Spain; 5 Hospital Son Espases, 07120 Palma de Mallorca, Spain; ines.legarda@ssib.es; 6 Hospital del Bierzo, 24404 Ponferrada, Spain; tagonfer@hotmail.com; 7 Hospital Universitario La Paz, 28046 Madrid, Spain; anarods@hotmail.es; 8 Complejo Hospitalario Universitario de Toledo, 45007 Toledo, Spain; mabelmc3006@gmail.com; 9 Hospital de Sant Joan Despí Moises Broggi, 08970 Barcelona, Spain; aperalq@csi.cat (A.P.); nuriacaballol@hotmail.com (N.C.); 10 Hospital Universitario de Elche, 03203 Elche, Spain; mariaalsa@hotmail.com; 11 Hospital de Galdakao Usansolo, 48960 Galdakao, Spain; iriacamro@gmail.com; 12 Hospital Universitario Nª Sª de Candelaria, 38010 Santa Cruz de Tenerife, Spain; deborahalonsomodino@gmail.com; 13 Hospital La Princesa, 28006 Madrid, Spain; lydialopez@hotmail.com; 14 Hospital Universitario de Torrecárdenas, 04009 Almería, Spain; olivares.je@gmail.com; 15 Hospital Universitario Juan Ramón Jiménez, 21005 Huelva, Spain; ablancoollero@telefonica.net

**Keywords:** effectiveness, foslevodopa/foscarbidopa, levodopa-entacapone-carbidopa, Parkinson’s disease, safety

## Abstract

**Highlights:**

**What are the main findings?**

- The reasons for the change and the outcome of patients treated with fLD/fCD who switched to LECIG are described in detail for the first time.
- The response to LECIG was favorable, with significant reduction of “Off” time and improvement in motor symptoms with a trend towards improvement in quality of life and NMS burden, as well as an adequate tolerability profile.

**What are the implications of the main findings?**

- LECIG could be an alternative therapeutic option in PwP who failed fLD/fCD.
- These findings reinforce the idea of the possibility of switching or associating DATs when the response to a previous DAT is not satisfactory.

**Abstract:**

**Introduction:** The clinical outcome of switching to levodopa-entacapone-carbidopa intestinal gel (LECIG) after failure of subcutaneous foslevodopa/foscarbidopa (fLD/fCD) is unknown. We analyze it in people with Parkinson’s disease (PwP) treated in Spain. **Methods:** Retrospective analysis of PwP who had previously received fLD/fCD but dropped out for different reasons and started before this LECIG in Spain up to 30 November 2025. Non-parametric tests were applied to evaluate the changes between the pre- (Vpre) and post-treatment (Vpost) (LECIG) periods. **Results:** Data about 14 patients (57.1% males; 66.6 ± 8.6 years old) from 12 hospitals out of a total of 15 who were treated with LECIG were included. The mean time with fLD/fCD was 98.6 ± 92.3 days, with 92.9% and 57.1% experiencing side effects and lack of response, respectively. Specifically, significant subcutaneous nodules were reported in up to 64.3% of the patients. LECIG was a direct switch from fLD/fCD in 35.7% of the patients. LECIG was well tolerated, with only one dropout due to complications related to dementia. Adverse events were reported in 28.6% and 35.7% of the patients in the optimization and final follow-up evaluation (mean follow-up of 233.7 ± 157.4 days) phases, respectively. From Vpre to Vpost, “Off” time was reduced in 2.9 ± 1.9 h (*p* = 0.002) and motor symptoms burden improved significantly (*p* = 0.013), whereas a trend of significance was found for non-motor symptoms burden (*p* = 0.050) and quality of life (*p* = 0.126). **Conclusions:** LECIG could be an alternative therapeutic option in PwP who failed fLD/fCD.

## 1. Introduction

Device-aided therapies (DATs) in Parkinson’s disease (PD) are advanced treatment modalities used when oral or transdermal medications no longer provide adequate control of motor fluctuations or dyskinesias [1]. For many years, three DATs have been used in many countries: deep brain stimulation (DBS), levodopa-carbidopa intestinal gel (LCIG) infusion, and continuous subcutaneous apomorphine infusion (CSAI) [2]. However, new formulations such as foslevodopa-foscarbidopa (fLD/fCD), a prodrug-based subcutaneous infusion, and levodopa-carbidopa-entacapone intestinal gel (LECIG) have emerged with the aim to provide continuous dopaminergic stimulation and reduce device-related complications [3]. In particular, fLD/fCD offers a non-surgical, continuous, and adjustable alternative to existing DATs such as LCIG and DBS for patients with advanced PD and motor fluctuations not adequately controlled by oral medication. Clinical trials have demonstrated that continuous subcutaneous fLD/fCD significantly increases “On” time without troublesome dyskinesia and reduces “Off” time compared to oral immediate-release levodopa/carbidopa, with benefits observed as early as the first post-baseline assessment and sustained over 12 weeks [4]. Given its ease of implementation and the good profile of levodopa in terms of safety and effectiveness, fLD/fCD is a good DAT option in many patients with PD, being the most frequently used option currently in Spain [5,6]. However, adverse events such as infusion site reactions (erythema, pain, cellulitis, edema), which are generally mild to moderate and manageable with site rotation and aseptic technique, or others related to the drug (visual hallucinations, psychosis, etc.) may lead to discontinuation of the treatment in some cases [4,7]. LECIG can be an alternative option for people with PD (PwP) who failed fLD/fCD, and in fact, a prospective, international, non-interventional phase IV study (SWITCH-ON) designed to assess the effectiveness of LECIG on the reduction in “Off” time from baseline at 12 months in PwP who failed fLD/fCD, is ongoing.

To date, no data has been published on the outcome of patients with PD treated with fLD/fCD who switch to LECIG. Here, we report our experience in Spain with PwP treated with LECIG who had previously failed subcutaneous fLD/fCD.

## 2. Materials and Methods

PwP treated with LECIG in Spain until 30 November 2025, who previously failed fLD/fCD, were included in this multicenter, longitudinal, retrospective, observational study. An in-person visit (post-LECIG) was conducted in different centers of Spain from 1 December 2025, to 10 January 2026, to assess patients already receiving LECIG and to collect data on their outcome. During this visit, the patient signed the informed consent form. Information on sociodemographic aspects, comorbidity, factors related to PD, and treatment including levodopa equivalent daily dose (LEDD) [8], fLD/fCD failure, and LECIG indication, was retrospectively collected.

The change from pre-LECIG (Vpre) to post-LECIG (Vpost) in the mean daily “Off” time, Unified Parkinson’s Disease Rating Scale (UPDRS) part III, health-related quality of life (PDQ-39 [39-item Parkinson’s Disease Questionnaire] total score), motor symptoms burden, and non-motor symptoms (NMS) burden were assessed. Specifically, Vpre was considered to be at the time of LECIG indication by the neurologist (before starting therapy), patients in some cases were on fLD/fCD (direct switch to LECIG) or others were on oral medication (previous abandonment). Vpost was considered to be when the patient was already receiving LECIG, with a variable time depending on the visit made and the start date. To assess motor symptoms burden, a motor symptoms score (MSs) [5,6] with a range from 0 to 38 was calculated, being the result of the sum of the score of “daily Off time” (from 0 to 4 according to the UPDRS part IV—item 39) + “daily dyskinesia time” (from 0 to 4 according to the UPDRS part IV—item 32) + other motor symptoms (dyskinesia severity; painful dyskinesia; morning dystonia; freezing of gait during the “Off” state; freezing of gait during the “On” state; falls; posture; tremor; hypomimia; speech problems) that were scored as 0 (none), 1 (slight), 2 (moderate) or 3 (severe) (Table S1-Supplementary Material). Regarding NMS burden, the same methodology was applied with a NMS score (NMSs) [5,6] from 0 to 54 that was the result of the sum of the score of different NMS, from 0 (none) to 3 (severe) (non-motor fluctuations; cognitive impairment; visual hallucinations; psychosis; impulse control disorder; depression; anxiety; apathy; sleep behavior disorder; diurnal somnolence; urinary symptoms; gastrointestinal symptoms; symptomatic orthostatic hypotension; constipation; sialorrhea; dysphagia; fatigue; pain) (Table S1-Supplementary Material). The data for MSs and NMSs at Vpre was obtained from a chart review. Data about the impression of change with fLD/fCD and with LECIG (post-LECIG) was collected according to the Clinical Global Impression of Change (CGI-C) scale. Adverse events were also collected.

Data were processed using SPSS 20.0 for Windows. Different variables were expressed as quantitative and/or qualitative variables. Distribution for variables was verified by one-sample Kolmogorov–Smirnov test. The change from pre-LECIG to post-LECIG in the mean daily “Off” time, PDQ-39 total score, MSs, and NMSs was assessed using the Wilcoxon rank-sum test. The marginal homogeneity test was used to compare the CGI-C with f/LD/fCD vs. with LECIG. Values of *p*  <  0.05 were considered significant.

## 3. Results

Data about 14 patients (57.1% males; 66.6 ± 8.6 years old) from 12 hospitals in Spain out of a total of 15 who were treated with LECIG were included. All patients received a 24 h/day continuous subcutaneous infusion of fLD/fCD with a mean exposure time of 98.6 ± 92.3 days, with a range from 20 to 301. The frequency of use of other dopaminergic drugs associated with fLD/fCD was 28.6% for MAO-B inhibitors, 21.4% for amantadine, 14.3% for COMT inhibitors, and 7.1% for dopamine agonists. Regarding the cause of fLD/fCD failure, up to 92.9% and 57.1% of the patients experienced side effects and lack of responses, respectively. Specifically, significant subcutaneous nodules with/without other skin problems were reported in up to 64.3% of patients (in eight patients it was the main reason to switch to LECIG), whereas visual hallucinations with psychotic symptoms were in 28.6% (in two patients it was the reason to switch to LECIG). Five patients (35.7%) received fLD/fCD for more than 3 months, with nodules and other skin problems being the major reason to switch to LECIG in four of them.

LECIG was a direct switch from fLD/fCD in 35.7% of the patients. At the time LECIG was indicated by the neurologist, the mean time from diagnosis of PD was 13.4 ± 8.2 years, and 100% of the patients had motor fluctuations and non-motor fluctuations with a mean daily time during the “Off” state of 5.2 ± 3.0 h. Patients had a good response to levodopa (levodopa challenge test) from the “Off” to the “On” state (Hoeh&Yahr stage and UPDRS part III), with 50% of them presenting cognitive impairment and 28.6% psychosis (Table 1). In addition to fLD/fCD, one patient had previously been treated with CSAI and two with LCIG. LECIG was initiated with hospitalization in more than half of the cases (57.1%), with the average optimization time being 14.9 ± 24.1 days (Table 1). At LECIG initiation, the mean cartridge volume (mL) used was 56.6 ± 15.9, and LEDD was significantly lower than at LECIG indication (1664.6 ± 449.0 vs. 1992.1 ± 689.9; *p* = 0.019) and at fLD/fCD withdrawal (1664.6 ± 449.0 vs. 2084.9 ± 726.3; *p* = 0.030).

**Figure 1:**
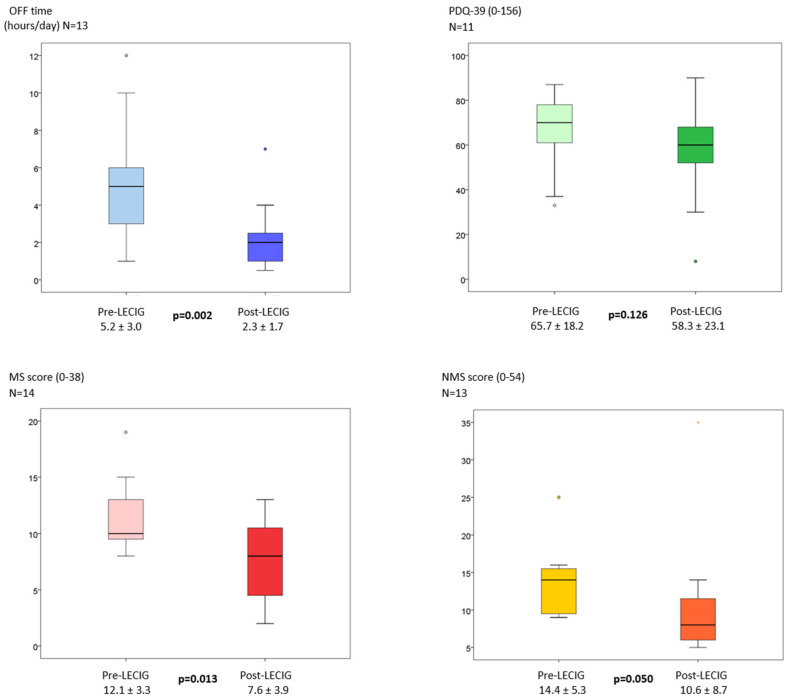
Change from pre-LECIG to post-LECIG in the mean daily “Off” time (hours), PDQ-39 (quality of life, 0–156), motor symptoms burden (MS score, 0–38), and non-motor symptoms burden (NMS score, 0–54). Data are presented as box plots, with the box representing the median and the two middle quartiles (25–75%). Mild outliers (O) are data points that are more extreme than Q1–1.5. Nonparametric tests were applied. NMSs, non-motor symptoms score; MSs, motor symptoms score.

The mean exposure time to LECIG (from Vpre to Vpost) was 233.7 ± 157.4 days. Daily OFF time was significantly reduced by 2.9 ± 1.9 h (*p* = 0.002), from 5.2 ± 3 at Vpre to 2.3 ± 1.7 at Vpost (Figure 1). PwP improved significantly from Vpre to Vpost in motor symptoms, with a decrease in the MSs from 12.1 ± 3.3 to 7.6 ± 3.9 points (−37.2%; *p* = 0.013). Regarding NMS burden, a trend of significance was detected in their improvement with a decrease in the NMSs from 14.4 ± 5.3 points at Vpre to 10.6 ± 8.7 at Vpost (−35.8%; *p* = 0.050). In terms of quality of life, the total score in the PDQ-39 from Vpre to Vpost was reduced by 7.4 points, but it was not statistically significant (Figure 1). No significant change was detected in the mean LEDD (mg) from Vpre (1664.6 ± 449.0) to Vpost (1718.0 ± 468.6) (*p* = 0.508). No differences were detected either (*p* = 0.726) between the UPDRS part III in the “On” state at Vpre (19.2 ± 16.8) and at Vpost (18.3 ± 16.0). Weight remained stable between both visits (70.5 ± 15.3 at Vpre vs. 69.8 ± 16.4 at Vpost; *p* = 0.505). The clinical perception of improvement experienced by patients based on the CGI-C was significantly better in the case of LECIG compared to fLD/fCD according to the neurologist’s (*p* = 0.017) and the patient’s own opinion (*p* = 0.012), but not in the case of the caregiver (*p* = 0.072) (Figure S1-Supplementary Material).

LECIG was well tolerated, with only one dropout (7.1%) due to complications related to dementia (the Vpost data was also collected in this case). Adverse events were reported in 28.6% and 35.7% of the patients in the optimization and final follow-up evaluation phases, respectively. Specifically, in the LECIG optimization phase, the following adverse events were recorded: stoma infection (N = 2), stoma erythema (N = 2), dyskinesia impairment (N = 2), tube migration (N = 1) and problems with gastrostomy (N = 1). In global terms, a total of 20 adverse events were reported, with complications in the stoma (N = 11) being the most frequent (Table 2). Fifty percent of the patients suffered from at least one possibly related (LECIG and/or device) adverse event.

## 4. Discussion

The present study observed a favorable outcome in the medium term (near 8 months on average) of PwP treated with LECIG who had previously failed fLD/fCD. In particular, a reduction in “Off” time and improvement in motor symptoms were found, with a trend towards improvement in quality of life and NMS burden. The tolerability profile of LECIG was adequate, in line with previous studies [9,10,11]. Despite the small sample size and pending data from the SWITCH-ON study, this is the first report on the outcome of patients treated with LECIG who previously failed fLD/fCD.

Most patients from our cohort who dropped fLD/fCD did so because of side effects, including more than 60% with nodules that in many cases were associated with a suboptimal response due to probable problems with drug absorption. Importantly, an average time of more than 3 months with fLD/fCD suggests a clear effort by the neurologists to try to optimize the patient prior to changing therapy. The frequency of skin problems in patients treated with fLD/fCD is high, with 62% of patients experiencing infusion/catheter site reactions and 28% experiencing infusion/catheter site infections in phase 3 clinical trials [12]. The most common skin reactions include erythema (27%), pain (26%), cellulitis (19%), edema (12%), bruising (3%), and hemorrhage (3%). These reactions are generally mild to moderate but can lead to treatment discontinuation in a subset of patients (8% for site reactions, 5% for site infections) [4,7,12,13]. The development of hallucinations or psychotic events can also be a reason for therapeutic failure in 15% of the patients [4], as in 14.3% of our patients. The global frequency of fLD/fCD withdrawal in clinical practice is at least 20% during the initial 12 weeks of therapy [4,7], with adverse events—primarily skin reactions—occurring in up to 87% of patients. When fLD/fCD fails, DATs are the main alternatives for advanced disease refractory to oral and adjunctive pharmacotherapy. CSAI would not be recommended in a patient with psychosis or pre-existing skin problems [14]. Alternatively, enteral levodopa infusion could be considered in these cases, with LECIG providing an equivalent efficacy to LCIG with a lower levodopa dose, similar safety and tolerability, and practical device advantages due to the addition of entacapone [15]. Data from previous studies on the use of DATs in Spain have shown that LECIG is indeed a frequently used therapeutic option when other DATs have previously failed [5,6]. But even in these cases, many patients can have a favorable response, with improvement in motor and NMS and maintenance of therapy [9,10,11]. In line with the findings observed in the ELEGANCE and LECIPARK studies, patients from this cohort reduced “Off” time by nearly 3 h and improved motor symptoms, with a trend towards improvement in NMS and their quality of life. In fact, the reduction of 7.4 points on the PDQ-39 total score is equivalent to 4.7 points on the PDQ-39SI, which is exactly what has been defined in the literature as the minimal clinically important difference [16]. It is noteworthy that these are patients with an advanced stage of the disease who did not improve satisfactorily with fLD/fCD. In this regard, there is data suggesting that patients who switch from LCIG to LECIG and are not optimally controlled may also improve [9]. The addition of entacapone increases levodopa bioavailability, allowing for a 20–35% reduction in the levodopa maintenance dose while achieving similar systemic exposure and motor response as LCIG, regardless of COMT genotype [17]. Importantly, the improvement achieved with LECIG in this cohort occurred without an increase in the LEDD. Differences in the route of administration or in the product itself could explain achieving improvement without increasing the LEDD. In this context, and although treatment should always be individualized based on aspects such as motor and non-motor phenotype, comorbidities and lifestyle factors, and of course, the opinion of the patient and their family, in general, for patients eligible for a DAT other than deep brain stimulation, and based on costs, invasiveness and ease of implementation, the subcutaneous route would be preferred over gastrostomy, with LECIG being an alternative treatment option in patients with previous failure [5,6,18,19,20]. Switching between and combining DATs can in some patients bring substantial clinical improvement and should be considered in those who have either inadequate symptom control on DAT treatment or have developed DAT-related complications [21]. In this cohort, the strategy (direct vs. non-direct switch) was at the neurologist’s discretion under clinical practice conditions. It was a personal decision, likely influenced by the time required to implement a new therapy (i.e., LECIG) based on hospital resources, as well as tolerability to fLD/fCD and the need to discontinue it.

The present study has important limitations. First, the sample size is small. Second, the methodology is that of an open and retrospective study. Third, in the follow-up evaluation (Vpost) with LECIG, the data for the variables in which the change was measured were not present in all cases. Fourth, we used non-validated scores (MSs and NMSs) to calculate the change in motor and NMS. Fifth, underreporting of adverse events cannot be ruled out. From a statistical point of view, borderline findings (e.g., NMS improvement) should be interpreted cautiously given the small cohort and multiple comparisons with the potential risk of a type I error. Finally, the average follow-up time is almost 8 months, making it important to know the longer-term evolution. All these factors may limit the generalizability of the findings, and they should be interpreted with caution. On the other hand, and despite these limitations, the findings are novel since, pending the results of the ongoing SWITCH-ON study, no data have been previously reported on PwP treated with LECIG who failed fLD/fCD.

## 5. Conclusions

In conclusion, based on this first Spanish experience report, LECIG could be an alternative therapeutic option in PwP who failed fLD/fCD. Data from prospective multicenter studies with a larger number of patients are needed to corroborate this initial observation.

## Figures and Tables

**Table 1:** Data about sociodemographic aspects, comorbidities, antiparkinsonian drugs and other therapies regarding treatment with fLD/fCD, indication of LECIG, and initiation of LCIG (N = 14).

Males (%)	57.1	H&Y–OFF	4 [3,4.5]
		H&Y–ON	2 [1.5,2]
Principal caregiver (%)	78.6	UPDRS–III–OFF	39.8 ± 9.8
Civil status (%):		UPDRS–III–ON	19.2 ± 16.8
- Married	64.3	Dyskinesia (%)	92.8
- Widowed	14.3	Cognitive impairment (%):	50
- Divorced	14.3	- MCI (%)	42.9
- Other	7.1	- Dementia (%)	7.1
		Psychosis (%)	28.6
Living style (%)		Polyneuropathy (%)	7.1
- With the partner	35.7		
- With the partner and son/daughter	21.4	Entacapone previously (%)	64.3
- Institutionalized	14.3	On-demand therapy previously (%)	64.3
- Other	28.6	- Inhaled levodopa	50
		- Subcutaneous apomorphine	21.4
**Treatment with fLD/fCD**		- Sublingual apomorphine	7.1
- 24 h infusion (%)	100		
- Daily volume (mL)	10.4 ± 3.6	Another DAT previously (%):	
- Days receiving fLD/fCD	98.6 ± 92.3	- CSAI	7.1
- LEDD–fLD/fCD (mg)	1827.3 ± 681.8	- LCIG	14.3
- LEDD (all PD-treatment) (mg)	2084.9 ± 726.3		
- MAO-B inhibitor (%)	28.6	Treatment added to levodopa (%):	
- COMT inhibitor (%)	14.3	- Levodopa	
- Dopamine agonist (%)	7.1	- MAO-B inhibitor	50
- Amantadine (%)	21.4	- COMT inhibitor	50
- Complications/AEs related to fLD/fCD (%)	92.9	- Dopamine agonist	14.3
- Lack of effectiveness with fLD/fCD (%)	57.1	- Amantadine	21.4
		- LEDD (mg)	1992.1 ± 689.9
**Indication of LECIG**			
Age	66.6 ± 8.6	**Initiation of LECIG**	
		How LECIG was started (%):	
Decision about the DAT (%):		- Hospitalization	57.1
- Without fLD/fCD at LECIG initiation	64.3	- At the day hospital	21.4
- Direct switch from fLD/fCD to LECIG	35.7	- On an outpatient basis	21.4
		Days for full LECIG optimization	14.9 ± 24.1
Weight (kg)	70.5 ± 15.3	Morning dose (mL)	12.2 ± 5.6
Height (cms)	167.5 ± 9.3	Infusion rate F1 (mL/h)	2.7 ± 0.7
BMI	25.1 ± 4.8	Infusion rate F2 (mL/h)	2.9 ± 0.5
		Infusion rate F3 (mL/h)	2.0 ± 0.6
Time from diagnosis of PD (years)	13.4 ± 8.2	Extra dose (mL)	2.3 ± 0.6
Motor fluctuations (%)	100	Daily volume (mL)	56.6 ± 15.9
Time with motor fluctuations (years)	5.5 ± 3.8	LEDD–LECIG (mg)	1506.3 ± 423.9
Non-motor fluctuations (%)	100	LEDD (all PD-treatment) (mg)	1664.6 ± 449.0
Daily “Off” time (hours) *	5.2 ± 3.0	Complications at LECIG initiation (%)	28.6

The results represent % or mean ± SD. *, N = 13. LEDD–fLD/fCD and LEDD–LECIG refer to LEDD provided only with the DAT (calculation based on total mL per day). BMI, body mass index; COMT, catechol-O-methyl transferase; CSAI, continuous subcutaneous apomorphine infusion; DAT, device-aided therapy; DBS, deep brain stimulation; H&Y, Hoehn&Yahr; LCIG, levodopa-carbidopa infusion gel; LECGI, levodopa-entacapone-carbidopa infusion gel; LEDD, levodopa equivalent daily dose; MCI, mild cognitive impairment; UPDRS, Unified Parkinson’s Disease Rating Scale.

**Table 2:** Adverse events collected by the neurologist in patients receiving LECIG (N = 14).

	N
Total AEs, N	20
- Stoma infection	4
- Stoma erythema	4
- Granuloma	3
- Tube migration	2
- Dyskinesia impairment	2
- Significant weight loss	2
- Problems with gastrostomy	1
- Orthostatic hypotension/hypotension	1
- Cognitive impairment	1
- Trauma due to fall with subdural hematoma	1
Patients with at least one AE, N (%)	7 (50)
At least possibly related (LECIG and/or device) AEs, N	18
Patients with at least possibly related (LECIG and/or device) AEs, N (%)	7 (50)
Patients with at least one AE leading to discontinuation, N (%)	1 (7.1)
- Cognitive impairment (to dementia and general deterioration)	
Patients with at least one possibly related (LECIG and/or device) leading to discontinuation N (%)	0 (0)
Deaths, N (%)	0 (0)

The results represent N or N (%). AEs, adverse events.

## Data Availability

The protocol, statistical analysis plan and unidentified participant data will be available on request due to privacy reasons. In any case, the protocol, statistical analysis plan, and unidentified participant data will be available on request.

## Supplementary Materials

The following supporting information can be downloaded at: https://www.mdpi.com/article/10.3390/brainsci16030343/s1, Figure S1: Clinical Impression Global of Change according to the opinion of the neurologist, patient, and principal caregiver with fLD/fCD (A) and with LECIG (B) (N = 14). P was significant for neurologist’s (*p* = 0.017) and the patient’s own opinion (*p* = 0.012), but not in the case of the caregiver (*p* = 0.072). The marginal homogeneity test was used. CGI-C, Clinical Global Impression of Change; fLD/fCD, foslevodopa/foscarbidopa; LECIG, levodopa-carbidopa-entacapone intestinal gel. Table S1. MSs and NMSs used for this study.

## Abbreviations

The following abbreviations are used in this manuscript:

CGI–C	Clinical Global Impression of Change
CSAI	continuous subcutaneous apomorphine infusion
DATs	device-aided therapies
DBS	deep brain stimulation
fLD/fCD	foslevodopa/foscarbidopa
LCIG	levodopa-carbidopa intestinal gel infusion
LECIG	levodopa-carbidopa-entacapone intestinal gel
LEDD	levodopa equivalent daily dose
MSs	motor symptoms score
NMS	non-motor symptoms
NMSs	non-motor symptoms score
PDQ-39	39-item Parkinson’s Disease Questionnaire
PD	Parkinson’s disease
PwP	people with PD
UPDRS	Unified Parkinson’s Disease Rated Scale
