# Supplementary material for: Outcome of People with Parkinson’s Disease Treated with Levodopa-Entacapone-Carbidopa Intestinal Gel Who Failed Previous Subcutaneous Foslevodopa/Foscarbidopa
Source: Brain Sci. 2026 Mar 22;16(3):343. doi: 10.3390/brainsci16030343 (PMC13024195; doi:10.3390/brainsci16030343)
Supplement: Supplementary file 1 [file brainsci-16-00343-s001.zip › Figure S1.SM.LECIDUOPARK.pdf]

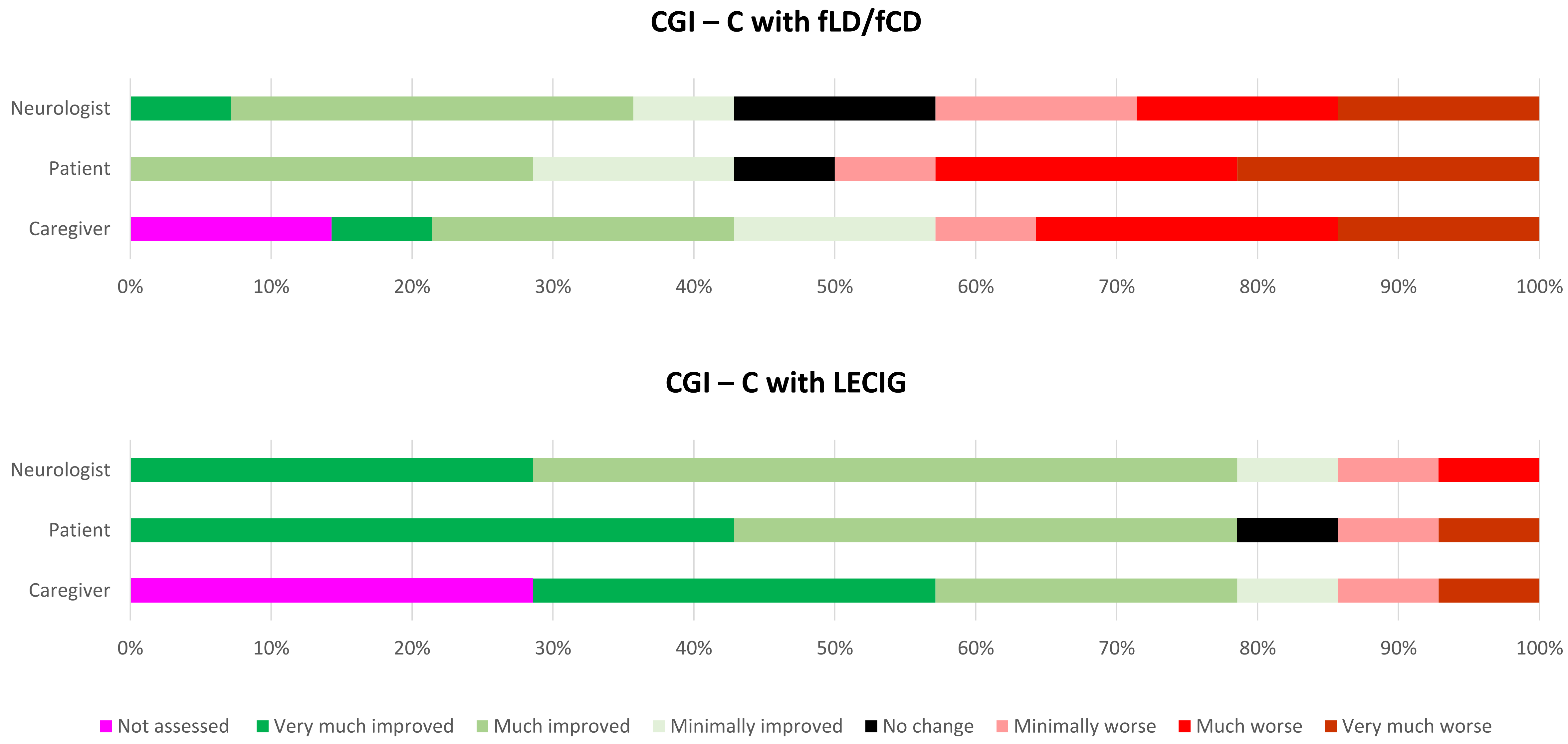
CGI – C with fLD/fCD
Neurologist
Patient
Caregiver
0%
10%
20%
30%
40%
50%
60%
70%
80%
90%
100%
CGI – C with LECIG
Neurologist
Patient
Caregiver
0%
10%
20%
30%
40%
50%
60%
70%
80%
90%
100%
Not assessed
Very much improved
Much improved
Minimally improved
No change
Minimally worse
Much worse
Very much worse

Figure S1.SM
