# Supplementary material for: Outcome of People with Parkinson’s Disease Treated with Levodopa-Entacapone-Carbidopa Intestinal Gel Who Failed Previous Subcutaneous Foslevodopa/Foscarbidopa
Source: Brain Sci. 2026 Mar 22;16(3):343. doi: 10.3390/brainsci16030343 (PMC13024195; doi:10.3390/brainsci16030343)
Supplement: Supplementary file 1 [file brainsci-16-00343-s001.zip › Table S1.SM.pdf]

Table S1. SM. MSs and NMSs used for this study.

Motor symptoms score (**MSs**) is the result of the sum of each item, from 0 (12 x 0) to 38 (4 + 4 + [10 x 3]).

Daily Off time	0 (0%)	1 (1-25%)	2 (26-50%)	3 (51-75%)	4 (76-100%)
Daily dyskinesia time	0 (0%)	1 (1-25%)	2 (26-50%)	3 (51-75%)	4 (76-100%)
Dyskinesia severity	0 (none)	1 (slight)	2 (moderate)	3 (severe)	
Painful dyskinesia	0 (none)	1 (slight)	2 (moderate)	3 (severe)	
Morning dystonia	0 (none)	1 (slight)	2 (moderate)	3 (severe)	
freezing of gait during the "Off" state	0 (none)	1 (slight)	2 (moderate)	3 (severe)	
freezing of gait during the "On" state	0 (none)	1 (slight)	2 (moderate)	3 (severe)	
falls	0 (none)	1 (slight)	2 (moderate)	3 (severe)	
Posture	0 (none)	1 (slight)	2 (moderate)	3 (severe)	
Tremor	0 (none)	1 (slight)	2 (moderate)	3 (severe)	
Hypomimia	0 (none)	1 (slight)	2 (moderate)	3 (severe)	
Speech problems	0 (none)	1 (slight)	2 (moderate)	3 (severe)	

Non-motor symptoms score (**NMSs**) is the result of the sum of each item, from 0 (18 x 0) to 54 (18 x 3).

Non-motor fluctuations	0 (0%)	1 (1-25%)	2 (26-50%)	3 (51-75%)
Cognitive impairment	0 (0%)	1 (1-25%)	2 (26-50%)	3 (51-75%)
Visual hallucinations	0 (none)	1 (slight)	2 (moderate)	3 (severe)
Psychosis	0 (none)	1 (slight)	2 (moderate)	3 (severe)
Impulse control disorder	0 (none)	1 (slight)	2 (moderate)	3 (severe)
Depression	0 (none)	1 (slight)	2 (moderate)	3 (severe)
Anxiety	0 (none)	1 (slight)	2 (moderate)	3 (severe)
Apathy	0 (none)	1 (slight)	2 (moderate)	3 (severe)
Sleep behavior disorder	0 (none)	1 (slight)	2 (moderate)	3 (severe)
Diurnal somnolence	0 (none)	1 (slight)	2 (moderate)	3 (severe)
Urinary symptoms	0 (none)	1 (slight)	2 (moderate)	3 (severe)
Gastrointestinal symptoms	0 (none)	1 (slight)	2 (moderate)	3 (severe)
Symptomatic orthostatic hypotension	0 (none)	1 (slight)	2 (moderate)	3 (severe)
Constipation	0 (none)	1 (slight)	2 (moderate)	3 (severe)
Sialorrhea	0 (none)	1 (slight)	2 (moderate)	3 (severe)
Dysphagia	0 (none)	1 (slight)	2 (moderate)	3 (severe)
Fatigue	0 (none)	1 (slight)	2 (moderate)	3 (severe)
Pain	0 (none)	1 (slight)	2 (moderate)	3 (severe)
